# Distances between mandibular posterior teeth and the WALA ridge in Peruvians with normal occlusion

**DOI:** 10.1590/2177-6709.22.6.056-060.oar

**Published:** 2017

**Authors:** Carla Y. Kong-Zárate, Marcos J. Carruitero, Will A. Andrews

**Affiliations:** 1 Private practice, Trujillo, Peru.; 2 Antenor Orrego Private University, School of Stomatology, Trujillo, Peru.; 3 Private practice, San Diego, California, USA.

**Keywords:** Mandibular posterior teeth, WALA ridge, Peruvian, Normal occlusion

## Abstract

**Objective:**

The purposes of this investigation were to determine the horizontal distances between the mandibular posterior teeth and the WALA ridge in a sample of Peruvians with normal occlusion and to compare them by tooth type, sex, arch side, and age groups.

**Methods:**

65 dental casts of subjects with normal occlusion were collected. Posterior teeth, except for third molars, were evaluated. The horizontal distances between the occluso-gingival midpoints of the buccal surfaces (FA points) of each tooth and the WALA ridge were measured using a modified digital caliper. The values between each different tooth type within the sample were compared using the ANOVA and Scheffe tests, while comparisons by sex, arch side and age groups, using the Student’s t-test.

**Results:**

The mean distances in the sample was 0.96 mm for first premolars, 1.45 mm for second premolars, 2.12 mm for first molars and 2.55 mm for second molars. Statistically significant differences between each of the four tooth types were found. There were no significant differences found between sex, arch side and age groups.

**Conclusion:**

The horizontal distances between the mandibular posterior teeth and the WALA ridge increased progressively from the first premolars to the second molars in Peruvians with normal occlusion. The WALA ridge was a good landmark to evaluate the positions of posterior teeth in Peruvians with normal occlusion.

## RESUMO

**Objetivos:**

o objetivo dessa investigação foi determinar a distância horizontal entre os dentes posteroinferiores e a borda WALA, em uma amostra de peruanos com oclusão normal, e compará-la por tipo de dente, sexo, lado da arcada dentária e grupo etário.

**Métodos:**

foram selecionados 65 modelos dentários de indivíduos com oclusão normal, nos quais foram avaliados os dentes posteriores, com exceção dos terceiros molares. As distâncias horizontais entre o ponto oclusogengival médio da face vestibular (pontos EV) de cada dente e a borda WALA foram medidas utilizando-se um compasso digital modificado. Os testes de Scheffe e ANOVA foram usados para comparações entre os valores de cada tipo de dente estudado, enquanto o teste *t* de Student foi usado para as comparações entre sexos, lados da arcada e faixa etárias.

**Resultados:**

as distâncias médias nessa amostra foram de 0,96 mm para os primeiros pré-molares; 1,45 mm para os segundos pré-molares; 2,12 mm para os primeiros molares, e 2,55 mm para os segundos molares. Foram encontradas diferenças estatisticamente significativas entre cada um dos quatro tipos dentários. Não houve diferenças significativas entre os sexos, lados da arcada e grupos etários.

**Conclusão:**

nos peruanos com oclusão normal dessa amostra, as distâncias horizontais entre os dentes posteroinferiores e a borda WALA aumentaram progressivamente dos primeiros pré-molares para os segundos molares. Nesses pacientes, a borda WALA funcionou como um bom ponto de referência para se avaliar a posição dos dentes posteroinferiores.

## INTRODUCTION

In orthodontic therapy the width and shape of the dental arch are key considerations.^1^ ^-^ ^3^ Because of the wide variety in dental arch forms between individuals, it is important to seek universal points of reference that could establish a guideline for individualizing arch forms for patients seeking orthodontic treatment.^2^ ^,^ ^4^ It has been suggested to use the buccal aspect of mandibular alveolar bone, known as the WALA ridge, as such a reference. The WALA ridge is clinically identifiable as a soft-tissue band immediately superior to the mucogingival junction.^5^ ^,^ ^6^ The supero-inferior position of the WALA ridge approximates that of the horizontal centers-of-rotation of teeth and has been shown to reliably relate to the shape of the underlying basal bone of the mandible.^7^ As such, this anatomic landmark has the potential to serve as a diagnostic guideline for positioning the teeth relative to mandibular basal bone.^1^ ^,^ ^7^ ^-^ ^9^

It has been shown that the shape of dental arches must be individualized in order to account for individual anatomic variations.^2.10^ The use of generic templates derived from facial type or geometric formulas to determine the ideal form of the arch is questionable and should be avoided.^11^ The individualization of arch shape is especially important when archwires with memory of form are employed.^12^ In pursuit of defining the anatomic characteristics of optimal arches, arches from untreated optimal occlusion have been studied by measuring the positional relationships between the FA points of the crowns and the WALA ridge. These positional relations have been shown to be useful guidelines for individualizing dental arch shape in the samples that have been studied, where the horizontal distances of FA to the WALA ridge increased progressively from incisors to second molars approximately from 0 to 2.5 mm,^1,6^ thus clinicians could use the WALA ridge from the initial casts to individualize the final archwires of their patients.

Because arch shape could vary between ethnic groups, further investigation of the relationship between the FA points and the WALA ridge is justified. In Peru, there is relative ethnic diversity characterized by regional concentrations of various ethnic groups in the coastal, mountain and jungle areas. The place of the present study was from the north of Peru and consisted of a mostly mixed race population with white phenotypic components of Mediterranean mixed with native-Americans characteristics. This ethnic mixture is found throughout the country, but particularly in the coastal area.^13^ There was no reported studies that have identified the horizontal distances of FA to the WALA ridge for this population, likewise it has not been investigated the comparison of these distances between females and males, right and left arch sides neither between young and adults.

This study aims to determine the horizontal distances between the mandibular posterior teeth and the WALA ridge in a sample of Peruvians with normal occlusion and to compare them by tooth types, sex, arch side and age groups.

## MATERIAL AND METHODS

The study protocol was approved by the Stomatology Permanent Research Committee of a Private University in Peru (*Universidad Privada Antenor Orrego*).

### Study sample

The present study was conducted on subjects between 13 and 25 years old with normal occlusion from a northern city in Peru. Dental casts of these 65 subjects (mean = 19.28 ± 3.85 years) consisting of 28 women (13 to 24 years old, mean 18.32 ± 3.62) and 37 men (13 to 25 years old, mean 20.00 ± 3.92) were evaluated. There were two age groups: 20 subjects under 18 years old (13 to 17 years old, mean 14.35 ± 1.23) and 45 subjects of 18 years and older (18 to 25 years old, mean 21.47 ± 2.24). All casts were collected from universities, health centers and schools from individuals who met the selection criteria. The inclusion criteria were: complete permanent dentition with or without third molars, with all teeth in occlusion, no previous orthodontic treatment, good buccal facial anatomy of the teeth, with no restorations, no previous buccal surgery that could have modified its normal anatomy, and with at least four from the six keys of the normal occlusion with no significant malposition of posterior teeth.

### Dental cast measurements

**Figure 1:**
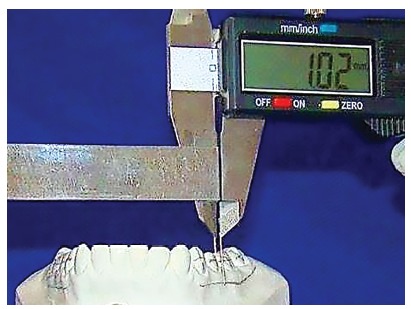
Measurements performed on casts showing the parallel position of the modified digital caliper to the occlusal plane. Pencil lines were drawn on the WALA ridge in the region of the four mandibular posterior teeth.

**Figure 2:**
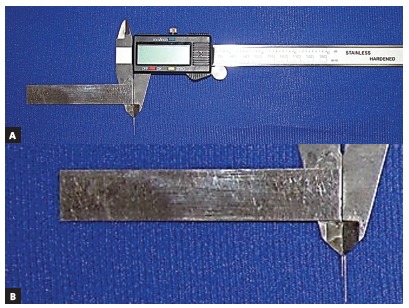
A) Modified digital caliper. B) Closer view of the main part of the modified caliper.

Pencil lines were drawn on the most facial aspect of the WALA ridge in the region of the four mandibular posterior teeth, from the first premolars to the second molars on each side of all mandibular dental casts (Fig 1). The occluso-gingival midpoint of the facial axis of the clinical crown (FACC), or FA point, was identified and marked with pencil on each mandibular posterior tooth, totaling 520 teeth. Finally, the horizontal distances between FA points and the WALA ridge were measured using a modified digital caliper (Fig 2). The measurements were made as parallel as possible to the occlusal plane.

### Method error

To evaluate the method error, measurements were repeated using the identical protocol for 10 randomly selected dental casts. These measurements were carried out by the same researcher twice (the second time after two weeks) in order to calculate intra-evaluator reliability. To assess the inter-evaluator reliability, the same random sample of 10 cases were evaluated by another researcher. Agreement between the measurements of the FA-WALA ridge distances for each posterior tooth were evaluated by the Concordance Correlation Coefficient test. 

### Statistical analysis

Data was processed in the statistical program Stata v. 12 (Stata Corp. Texas, USA). The means, standard deviations, and the minimum and maximum values were calculated. Before making any group comparisons, compliance with the assumptions of normality and homogeneity of variances with Shapiro-Wilk and Variance ratio test was evaluated. To compare the means in the groups that met the assumptions, ANOVA of repeated measures test and Scheffe *post-hoc* test were used. Comparisons between sex, and age groups were performed using Student’s t test for independent groups and between arch side using Student’s t-test for related groups. Statistical significance was set at 5% in all tests.

## RESULTS

Reliability was considered adequate. High concordance with values greater than 0.806 was found for inter and intra-evaluator evaluation (Table 1).

**Table 1:** Errors in the methods for FA-WALA ridge distances in mm (n=10).

Side	Mandibular posterior tooth	Calibration			
		Inter-evaluator		Intra-evaluator	
		CCC	P	CCC	p
Left	First premolar	0.831	<0.001	0.839	<0.001
	Second premolar	0.921	<0.001	0.961	<0.001
	First molar	0.836	<0.001	0.960	<0.001
	Second molar	0.871	<0.001	0.894	<0.001
Right	First premolar	0.939	<0.001	0.849	<0.001
	Second premolar	0.896	<0.001	0.887	<0.001
	First molar	0.891	<0.001	0.901	<0.001
	Second molar	0.806	<0.001	0.853	<0.001

CCC, Concordance correlation coefficient.

Table 2 shows comparisons according to sex, arch side and age groups. For all comparisons, there were no statistically significant differences between the groups (*p*> 0.05).

**Table 2:** Comparison of FA-WALA ridge distances (mm) in the Peruvian sample by sex, arch side and age groups.

Tooth	Sex					Arch side					Age (in years)				
	Female (n=28)		Male (n=37)		p*	Left (n=65)		Right (n=65)		p**	13-17 (n=20)		18-25 (n=45)		p*
	$\bar{\text{x}}$	SD	$\bar{\text{x}}$	SD		$\bar{\text{x}}$	SD	$\bar{\text{x}}$	SD		$\bar{\text{x}}$	SD	$\bar{\text{x}}$	SD	
1st premolar	0.92	0.38	0.99	0.35	0.139	0.94	0.36	0.97	0.38	0.322	0.99	0.39	0.94	0.36	0.239
2nd premolar	1.40	0.46	1.49	0.55	0.162	1.40	0.50	1.50	0.52	0.133	1.46	0.63	1.44	0.45	0.419
1st molar	2.04	0.57	2.18	0.61	0.093	2.04	0.59	2.20	0.59	0.062	2.20	0.68	2.09	0.55	0.165
2nd molar	2.49	0.61	2.58	0.60	0.201	2.64	0.56	2.47	0.66	0.058	2.56	0.65	2.55	0.61	0.337

* Student’s t-test for independent groups; ** Student’s t-test for relates groups; $\bar{\text{x}}$, mean; SD, standard deviation.

The average distance between the posterior mandibular teeth and alveolar process in the subjects evaluated was 0.96 mm for first premolars, 1.45 mm for second premolars, 2.12 mm for first molars, and 2.55 mm for second molars. Statistically significant differences were found between individual tooth types (*p*< 0.001) (Table 3). 

**Table 3:** FA-WALA ridge distances (mm) in the Peruvian sample.

Tooth	n	Mean‡	SD	Minimum	Maximum
First premolar	65	0.96	0.37	0.22	2.10
Second premolar	65	1.45	0.51	0.34	2.80
First molar	65	2.12	0.59	0.75	3.50
Second molar	65	2.55	0.62	1.02	3.95

‡General comparison by tooth type with ANOVA of repeated measures, F=229.72, p<0.001; *post-hoc* with Scheffe: p<0.001 to all comparisons; SD, standard deviation.

## DISCUSSION

The configuration of the dental arch varies widely among individuals. This is related to many factors, including dental alignment, tooth shape, size, musculature, facial patterns, jaw size and shape, cranial factors, and occlusion. This diversity has led some authors to recommend specific concepts for customizing the shape of dental arches for patients in order to improve the prospects of post-treatment stability, health, function, and appearance.^4^ ^-^ ^7^

One important goal of orthodontic therapy is to improve occlusion while positioning roots over supporting bone. A concept championed by Andrews utilizes the WALA ridge, an anatomical ridge on the facial aspect of the mandibular alveolar process as a landmark whose shape is correlated with the shape of the underlying basal bone.^5^ ^,^ ^6^ The use of an external anatomic landmark whose shape is unique for each individual patient allows the dental arch to be customized in a way that ensures that the roots of the teeth will be surrounded by alveolar bone and positioned over basal bone.^14^ Based on this concept, the study of the relationship between the teeth and the WALA ridge in an homogeneous sample is important, in order to establish standard distances between the FA points and the WALA ridge that could directly influence both treatment planning and the construction of customized archwires.

In the present study the dental casts of mestizo Peruvians from the north of the country with normal posterior occlusion were evaluated. The horizontal distances between the mandibular posterior teeth and WALA ridge followed a progressive pattern, increasing incrementally by roughly 0.5 millimeter from the first premolar to the second molar. These progressive increments were similar to those which have been previously reported by Brazilian^1^ and North American^6^ populations. 

When making comparisons by sex, no differences were observed, suggesting to consider this progressive increment equally for men and women. Similar interpretation correspond for adolescents and young adults after age groups comparison, coinciding with Gupta et al.^7^ whom found that dental and basal arch forms were not significantly different between adolescents and adults. Additionally, in the entire sample no difference between right and left sides were found. These findings are important because they show similar behavior of the distances between the WALA ridge and FA points in Peruvians and in other populations, to whom the WALA ridge is considered a good landmark to establish arch morphology.^2^ ^,^ ^7^

**Figure 3:**
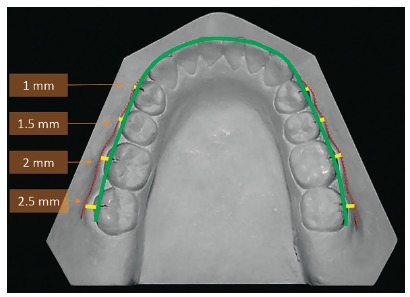
Representation of the individualized archwire (green line) constructed using the WALA ridge from the initial casts (red lines) and the FA-WALA ridge average distances (yellow lines).

Based on the results, the WALA ridge could be useful as a reference for determining the arch form, so the WALA ridge became a good landmark for assessing the facio-lingual of posterior teeth and could be used as a guide for constructing archwires. Similarly, Gupta et al.^7^ reported WALA points can be used to predict individual dental arch forms, also Ball et al.^15^ concluded that the use of the WALA points or other anatomic landmarks of the basal bone to predict the ideal dental arch form for a patient seems possible and could ensure a more stable orthodontic treatment outcome. In addition, Conti et al.^9^ showed that the WALA ridge allows individualization of the dental arches favoring the post-treatment stability. Thus, clinicians could use the FA-WALA ridge distances presented in this study and their progressive increase from first premolars to second molars as a reference from the initial casts to individualize the final archwires (Fig 3).

## CONCLUSIONS

» The mean horizontal distances between the mandibular posterior teeth (FA points) and alveolar process (WALA ridge) in the study subjects were 0.96 mm for first premolars, 1.45 mm for second premolars, 2.12 mm for first molars and 2.55 mm for second molars.

» Similar measures for men and women, age groups (13 to 17 and 18 to 25 years old), also for right and left sides were observed.

» The WALA ridge was a good landmark for assessing the facio-lingual positions of posterior teeth in Peruvians with normal occlusion, and can be used as a guide for constructing individualized archwires.
